# Synthesis and biological evaluation of benzenesulphonamide-bearing 1,4,5-trisubstituted-1,2,3-triazoles possessing human carbonic anhydrase I, II, IV, and IX inhibitory activity

**DOI:** 10.1080/14756366.2017.1367775

**Published:** 2017-09-11

**Authors:** Rajiv Kumar, Vikas Sharma, Silvia Bua, Claudiu T. Supuran, Pawan K. Sharma

**Affiliations:** a Department of Chemistry, Kurukshetra University, Kurukshetra, India;; b Neurofarba Department, Laboratorio di Chimica Bioinorganica, Sezione di Scienze Farmaceutiche, Università degli Studi di Firenze, Firenze, Italy

**Keywords:** 1,2,3-Triazoles, benzenesulphonamide, carbonic anhydrase, isoforms I, II, IV, IX, acetazolamide

## Abstract

A library of benzenesulphonamides incorporating 1,2,3-triazole rings functionalised with ester, carboxylic acid, carboxamide, carboxyhydrazide, and hydroxymethyl moieties were synthesised. The carbonic anhydrase (CAs, EC 4.2.1.1) inhibitory activity of the new compounds was assessed against four human (h) isoforms, hCA I, hCA II, hCA IV, and hCA IX. Among them, hCA II and IV are anti-glaucoma drug targets, being involved in aqueous humour secretion within the eye. hCA I was inhibited with Ki’s ranging between 8.3 nM and 0.8737 µM. hCA II, the physiologically dominant cytosolic isoform, was excellently inhibited by these compounds, with Ki’s in the range of 1.6–9.4 nM, whereas hCA IV was effectively inhibited by most of them, with Ki’s in the range of 1.4–55.3 nM. Thirteen of the twenty sulphonamides were found to be excellent inhibitors of tumour associated hCA IX with Ki’s ≤ 9.5 nM. Many of the new compounds reported here showed low nM inhibitory action against hCA II, IV, and IX, isoforms involved in glaucoma and some tumours, making them interesting candidates for further medicinal chemistry/pharmacologic studies.

## Introduction

Carbonic anhydrases (CAs, EC 4.2.1.1) belong to family of zinc metalloenzymes found in variety of organisms, including higher vertebrates and humans^1–3^. As all the seven families of CAs known to date (α, β, γ, δ, ζ, η, and ɵ-class)^4–6^ are involved in the reversible hydration–dehydration of carbon dioxide and bicarbonate ions, therefore, play vital roles in various important physiological processes, such as respiration, electrolyte secretion in variety of tissues/organs, biosynthetic reactions (i.e. lipogenesis, glucogenesis, and ureagenesis), bone resorption, calcification, etc.^1^^,^^2^^,^^7^^,^^8^. However, several studies demonstrated that abnormal levels or activities of these enzymes have been associated with various human diseases. Out of the sixteen isoforms of human associated α-class of CAs, some isoforms are involved in pertinent pathologies, such as glaucoma^9^, epilepsy^10^, obesity^11^, altitude sickness,1 retinitis pigmentos1 cancer^12–15^, etc. Therefore, carbonic anhydrase inhibitors (CAIs) have applications as therapeutic agents, such as antiglaucoma, antiobesity, antidiuretic, antiepileptic, anti-altitude sickness, antipain, and anti-infective agents^1^^,^^16–20^. However, designing and synthesising isoform-selective inhibitors are a challenging task for obtaining a drug with minimum side effect.

**Figure 1.**
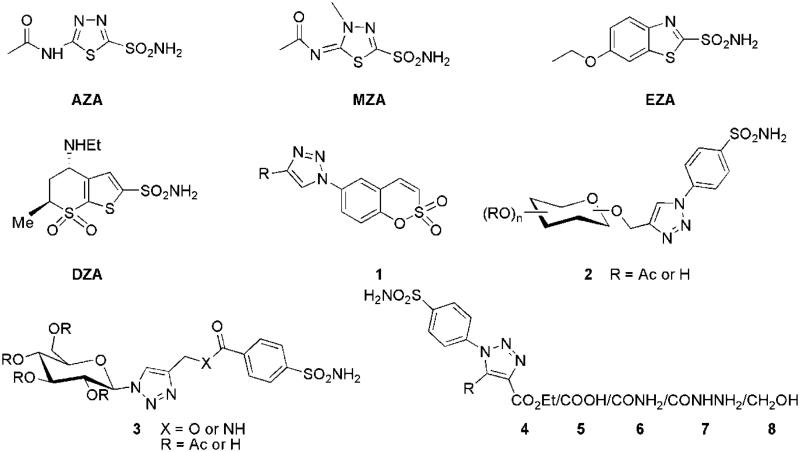
Some clinically used sulphonamide based drugs and 1,2,3-triazole ring containing CA inhibitors.

Primary sulphonamide bearing heterocyclic compounds form a part of potent CAIs in which binding group binds to the Zn(II) ion as anion in a tetrahedral geometry. A large number of drugs belonging to this class, like acetazolamide (AZA), methazolamide (MZA), ethoxzolamide (EZA), dorzolamide (DZA), etc., are in clinical use from past many years targeting different therapeutic areas^21^. Recently our research group has reported some fused 1,2,4-triazoles and 4-functionalised pyrazoles bearing benzenesulphonamide as selective inhibitors of CA IX and XII^22–24^. Further 1,2,3-triazole ring containing compounds are gaining interest in diverse therapeutic areas like antiproliferative,^25^ antitubercular, antimicrobial^26–28^, anticancer^29^^,^^30^, anti-HIV^31^, antifungal, antibacterial^32^, anti-inflammatory^33^, antiobesity^34^, antiviral^35^, etc., as well as in several DNA-alkylating, crosslinking agents^36^^,^^37^, and β-lactamase inhibitors^38^. Also some 1,2,3-triazole ring containing selective CAIs have been reported (**1**–**3**)^39^^,^^40^. Motivated by these findings and continuing our interest in the design of various classes of heterocyclic based compounds of potential medicinal interest^22–24^^,^^41–46^, we turned our attention towards the synthesis of a small library of novel 4-functionalised 1-aryl-5-alkyl/aryl-1,2,3-triazoles (**4a**–**4d**, **5a**–**5d**, **6a**–**6d**, **7a**–**7d**, and **8a**–**8d**) bearing a primary sulphonamide group on the phenyl ring at N-1 position of 1,2,3-triazole scaffold with different functionalities at C-4, such as ester, carboxylic acid, carboxamide, hydrazinocarbonyl, and hydroxymethyl (Figure 1) in order to investigate their carbonic anhydrases inhibition against isoforms hCA I, II, IV, and IX.

## Materials and methods

### General

All the commercially available chemicals were used without further purification. All the solvents were dried and/or purified according to standard procedures prior to use. All the reactions were monitored by thin layer chromatography (TLC) on TLC silica gel on F_254_ aluminium plates using a mixture of chloroform and methanol as eluent while UV lamp was used to visualise the spots. Melting points were determined in open capillaries in an electrical melting point apparatus and are uncorrected. IR spectra were recorded on ABB MB 3000 DTGS IR instrument using the KBr pellet technique. ^1^H NMR spectra were recorded on 400 MHz, while ^13^C NMR spectra were registered at 100 MHz, using deuterated dimethyl sulphoxide (DMSO-d_6_) as solvent, and tetramethylsilane (TMS) as internal standard at room temperature. Chemical shifts are reported as δ values in parts per million (ppm) downfield from TMS. High resolution mass spectra were obtained from a MicroMass ESI-TOF MS spectrometer. Multiplicities are described as singlet (s), doublet (d), doublet of doublet (dd), doublet of a doublet of a doublet (ddd), doublet of triplet (dt), triplet (t), quartet (q), multiplet (m), exchangeable proton (ex) for NMR assignments and strong (s), medium (m), broad (br) for IR assignments. The coupling constants are expressed in hertz (Hz).

### Synthesis of ethyl 1-[4-(aminosulfonyl)phenyl]-5-(alkyl/aryl)-1*H*-1,2,3-triazole-4-carboxylate (**4a**–**4d**)

General procedure: A mixture of appropriate β-diketoester **11a–11d** (2.00 mmol) and piperidine (5 mol%) dissolved in 3 ml DMSO were heated at 70 °C in silicon oil bath for 5 min followed by addition of 4-azidobenzenesulphonamide (2.02 mmol). After addition, reaction mixture was allowed to stir at 70 °C for 4–6 h. Reaction was monitored through thin-layer chromatography. After completion, reaction mixture was poured into water after cooling to afford required product **4a**–**4d**. The crude product thus obtained was recrystalised with ethanol.

### Ethyl 1-[4-(aminosulfonyl)phenyl]-5-methyl-1*H*-1,2,3-triazole-4-carboxylate (**4a**)

Yield 91%; white solid; mp: 212 °C; IR(KBr) (ν, cm^−1^): 3302, 3070 (m, N–H stretch), 2924 (m, –CH_3_ stretch), 1728 (s, C=O stretch),1335, 1165 (s, SO_2_stretch); ^1^H NMR (400 MHz, DMSO-d_6_) δ (ppm): 8.08 (dd, *J* = 8.8 Hz, *J* = 2.0 Hz, 2H, Ar), 7.88 (dd, *J* = 8.8 Hz, *J* = 2.0 Hz, 2H, Ar), 7.61 (s, 2H, SO_2_NH_2_), 4.37 (q, *J* = 7.2 Hz, 2H, CH_2_), 2.58 (s, 3H, –CH_3_), 1.35 (t, *J* = 7.2 Hz, 3H, CH_3_); ^13^C NMR (100 MHz, DMSO-d_6_) δ (ppm): 160.92, 145.31, 139.48, 137.47, 136.08, 127.14, 125.98, 60.50, 14.12, 9.73; HRMS (ESI-MS) *m/z* 333.0640 (M + Na)^+^, C_12_H_14_N_4_O_4_SNa^+^, calcd 333.0634.

### Ethyl 1-[4-(aminosulfonyl)phenyl]-5-phenyl-1*H*-1,2,3-triazole-4-carboxylate (**4b**)

Yield 75%; dirty white solid; mp: 184 °C; IR(KBr) (ν, cm^−1^): 3371, 3263, 3061 (m, N–H stretch), 1704 (s, C=O stretch), 1342, 1159 (s, SO_2_ stretch); ^1^H NMR (400 MHz, CDCl_3_) δ (ppm):): 7.96 (dd, *J* = 7.2 Hz, *J* = 1.2 Hz, 2H, Ar), 7.56–7.48 (m, 5H, Ar), 7.32 (dd, *J* = 8.4 Hz, *J* = 1.2 Hz, 2H, Ar), 7.06 (s, 2H, SO_2_NH_2_), 4.33 (q, *J* = 7.2 Hz, 2H, CH_2_), 1.28 (t, *J* = 7.2 Hz, 3H, CH_3_); ^13^C NMR (100 MHz, CDCl_3_) δ (ppm): 165.33, 149.56, 145.64, 142.84, 141.97, 135.02, 134.95, 133.38, 132.17, 130.08, 130.05, 65.94, 18.81; HRMS (ESI-MS) *m/z* 395.0785 (M + Na)^+^, C_17_H_16_N_4_O_4_SNa^+^, calcd 395.0789.

### Ethyl 1-[4-(aminosulfonyl)phenyl]-5-(4-methoxyphenyl)-1*H*-1,2,3-triazole-4-carboxylate (**4c**)

Yield 74%; pale yellow solid; mp: 176 °C; IR(KBr) (ν, cm^−1^): 3364, 3271 (m, N–H stretch), 2970 (m, –CH_3_ stretch), 1713 (s, C=O stretch), 1342, 1157 (s, SO_2_ stretch); ^1^H NMR (400 MHz, DMSO-d_6_) δ (ppm): 7.93 (dd, *J* = 6.8 Hz, *J* = 2.0 Hz, 2H, Ar), 7.59 (d, *J* = 8.8 Hz, 2H, Ar), 7.54 (s, 2H, SO_2_NH_2_), 7.32 (d, *J* = 8.8 Hz, 2H, Ar), 6.97 (dd, *J* = 6.8 Hz, *J* = 2.0 Hz, 2H, Ar), 4.23 (q, *J* = 7.2 Hz, 2H, CH_2_), 3.77 (s, 3H, –CH_3_), 1.18 (t, *J* = 7.2 Hz, 3H, CH_3_); ^13^C NMR (100 MHz, DMSO-d_6_) δ (ppm): 160.31, 160.26, 144.94, 141.09, 137.89, 136.28, 131.88, 126.82, 126.37, 116.99, 113.72, 60.46, 55.21, 13.89; HRMS (ESI-MS) *m/z* 403.1072 (M + H)^+^, C_18_H_18_N_4_O_5_SH^+^, calcd 403.1076.

### Ethyl 1-[4-(aminosulfonyl)phenyl]-5-(2-naphthyl)-1*H*-1,2,3-triazole-4-carboxylate (**4d**)

Yield 92%; dirty white solid; mp: 180 °C; IR(KBr) (ν, cm^−1^): 3325, 3240 (m, N–H stretch), 1728 (s, C=O stretch), 1335, 1165 (s, SO_2_ stretch); ^1^H NMR (400 MHz, DMSO-d_6_) δ (ppm): 8.07 (d, *J* = 8.4 Hz, 1H, Ar), 8.00 (d, *J* = 8.0 Hz, 1H, Ar), 7.78 (dd, *J* = 6.8 Hz, *J* = 2.0 Hz, 2H, Ar), 7.65 (dd, *J* = 7.2 Hz, *J* = 1.2 Hz, 1H, Ar),7.58 (d, *J* = 8.8 Hz, 3H, Ar), 7.54 (dd, *J* = 8.0 Hz, *J* = 1.2 Hz, 1H, Ar),7.48 (dt, *J* = 8.4 Hz, *J* = 1.6 Hz, 1H, Ar), 7.45–7.43 (m, 3H, Ar, SO_2_NH_2_), 4.04–4.00 (m, 2H, CH_2_), 0.81 (t, *J* = 7.6 Hz, 3H, –CH_3_); ^13^C NMR (100 MHz, DMSO-d_6_) δ (ppm): 159.80, 145.04, 139.66, 138.14, 137.71, 132.67, 131.10, 130.40, 129.42, 128.41, 127.33, 126.73, 126.42, 125.66, 125.06, 124.32, 123.12, 60.29, 13.25; HRMS (ESI-MS) *m/z* 423.1124 (M + H)^+^, C_21_H_18_N_4_O_4_SH^+^, calcd 423.1127.

### Synthesis of 1-[4-(aminosulfonyl)phenyl]-5-alkyl/aryl-1*H*-1,2,3-triazole-4-carboxylic acid (**5a**–**5d**)

General procedure: An appropriate 1,2,3-triazolic ester **4a**–**4d** was dissolved in 20% aq NaOH solution (5 ml) and refluxed for 4 h. Then cooled the solution to room temperature, added ice to it and neutralised with concd HCl which resulted into the precipitation of a white solid. The solid was filtered off, washed with water, dried and recrystallised from appropriate solvent which afforded the pure products **5a**–**5d**.

#### 1-[4-(Aminosulfonyl)phenyl]-5-methyl-1*H*-1,2,3-triazole-4-carboxylic acid (**5a**)

Yield 94%; white solid; mp: 198 °C; IR(KBr) (ν, cm^−1^): 3348, 3225 (m, N–H stretch), 3078 (br, O–H stretch), 2905 (m, –CH_3_ stretch), 1697 (s, C=O stretch),1334, 1150 (s, SO_2_ stretch); ^1^H NMR (400 MHz, DMSO-d_6_) δ (ppm): 13.24 (s, br, 1H, –COOH), 8.07 (d, *J* = 8.4 Hz, 2H, Ar), 7.87 (d, *J* = 8.4 Hz, 2H, Ar), 7.62 (s, 2H, SO_2_NH_2_), 2.56 (s, 3H, –CH_3_); ^13^C NMR (100 MHz, DMSO-d_6_) δ (ppm): 162.42, 145.24, 139.22, 137.63, 136.83, 127.16, 125.94, 9.74; HRMS (ESI-MS) *m/z* 305.0325 (M + Na)^+^, C_10_H_10_N_4_O_4_SNa^+^, calcd 305.0321.

#### 1-[4-(Aminosulfonyl)phenyl]-5-phenyl-1*H*-1,2,3-triazole-4-carboxylic acid (**5b**)

Yield 85%; white solid; mp: 156 °C; IR(KBr) (ν, cm^−1^): 3379, 3263 (m, N–H stretch), 3063 (br, O–H stretch),1713 (s, C=O stretch), 1342, 1165 (s, SO_2_ stretch); ^1^H NMR (400 MHz, DMSO-d_6_) δ (ppm): 12.66 (s, br, 1H, –COOH), 7.90 (dd, *J* = 6.8 Hz, *J* = 1.6 Hz, 2H, Ar), 7.59–7.57 (m, 4H, Ar, SO_2_NH_2_), 7.45–7.40 (m, 5H, Ar); ^13^C NMR (100 MHz, DMSO-d_6_) δ (ppm): 162.12, 145.41, 141.41, 138.33, 137.72, 130.84, 130.29, 128.71, 127.27, 126.92, 126.16; HRMS (ESI-MS) *m/z* 367.07473 (M + Na)^+^, C_15_H_12_N_4_O_4_SNa^+^, calcd 367.0477.

#### 1-[4-(Aminosulfonyl)phenyl]-5-(4-methoxyphenyl)-1*H*-1,2,3-triazole-4-carboxylic acid (**5c**)

Yield 98%; dirty white solid; mp: 148–150 °C; IR(KBr) (ν, cm^−1^): 3333, 3242 (m, N–H stretch), 3055 (br, O–H stretch), 2905 (m, –CH_3_ stretch), 1705 (s, C=O stretch), 1335, 1165 (s, SO_2_ stretch); ^1^H NMR (400 MHz, DMSO-d_6_) δ (ppm): 13.02 (s, br, 1H, –COOH), 7.90 (d, *J* = 8.8 Hz, 2H, Ar), 7.56 (d, *J* = 8.8 Hz, 2H, Ar), 7.55 (s, 2H, SO_2_NH_2_), 7.30 (d, *J* = 8.8 Hz, 2H, Ar), 6.95 (d, *J* = 8.8 Hz, 2H, Ar), 3.77 (s, 3H, –CH_3_); ^13^C NMR (100 MHz, DMSO-d_6_) δ (ppm): 161.69, 160.16, 144.85, 140.75, 137.98, 137.01, 131.85, 126.78, 126.32, 117.31, 113.72, 55.18; HRMS (ESI-MS) *m/z* 397.0577 (M + Na)^+^, C_16_H_14_N_4_O_5_SNa^+^, calcd 397.0582.

#### 1-[4-(Aminosulfonyl)phenyl]-5-(2-naphthyl)-1*H*-1,2,3-triazole-4-carboxylic acid (**5d**)

Yield 93%; pale yellow solid; mp: 198–200 °C; IR(KBr) (ν, cm^−1^): 3356, 3256 (m, N–H stretch), 3078 (br, O–H stretch), 1690 (s, C=O stretch), 1335, 1165 (s, SO_2_ stretch); ^1^H NMR (400 MHz, DMSO-d_6_) δ (ppm): 12.98 (s, br, 1H, COOH), 8.04 (d, *J* = 8.4 Hz, 1H, Ar), 7.97 (d, *J* = 8.0 Hz, 1H, Ar), 7.76 (dd, *J* = 8.8 Hz, *J* = 2.0 Hz, 2H, Ar), 7.66 (dd, *J* = 7.6 Hz, *J* = 1.2 Hz, 1H, Ar), 7.58–7.39 (m, 8H, Ar, SO_2_NH_2_); ^13^C NMR (100 MHz, DMSO-d_6_) δ (ppm): 161.28, 144.94, 139.32, 138.88, 137.83, 132.67, 131.06, 130.29, 129.48, 128.42, 127.30, 126.66, 126.40, 125.61, 125.11, 124.32, 123.39; HRMS (ESI-MS) *m/z* 395.0800 (M + H)^+^, C_19_H_14_N_4_O_4_SH^+^, calcd 395.0814.

### Synthesis of 1-[4-(aminosulfonyl)phenyl]-5-alkyl/aryl-1*H*-1,2,3-triazole-4-carboxamide (**6a**–**6d**)

General procedure: A solution of appropriate 1,2,3-triazolic ester **4a**–**4d** (1.29 mmol) in cold concentrated aq. ammonia solution (3 ml) was stirred for 22 h in a stoppered flask. The white precipitates of carboxamide derivatives **6a**–**6d** thus obtained were filtered, washed with excess of cold water, dried at 120 °C and recrystallised from ethanol.

#### 1-[4-(Aminosulfonyl)phenyl]-5-methyl-1*H*-1,2,3-triazole-4-carboxamide (**6a**)

Yield 74%; white solid; mp: 314–316 °C; IR(KBr) (ν, cm^−1^): 3456, 3356, 3271 (m, N–H stretch), 1674 (s, C=O stretch), 1333, 1173 (s, SO_2_ stretch); ^1^H NMR (400 MHz, DMSO-d_6_) δ (ppm): 8.06 (dd, *J* = 8.8 Hz, *J* = 2.0 Hz, 2H, Ar), 7.94 (s, ex, 1H, OH/NH), 7.87 (dd, *J* = 8.8 Hz, *J* = 2.0 Hz, 2H, Ar), 7.52–7.49 (m, ex, 3H, OH/NH, SO_2_NH_2_), 2.57 (s, 3H, –CH_3_); ^13^C NMR (100 MHz, DMSO-d_6_) δ (ppm): 162.59, 145.10, 138.47, 137.72, 137.01, 127.10, 125.80, 9.31; HRMS (ESI-MS) *m/z* 282.0656 (M + H)^+^, C_10_H_11_N_5_O_3_SH^+^, calcd 282.0661.

#### 1-[4-(Aminosulfonyl)phenyl]-5-phenyl-1*H*-1,2,3-triazole-4-carboxamide (**6b**)

Yield 76%; pale yellow solid; mp: 210 °C; IR(KBr) (ν, cm^−1^): 3479, 3379, 3178, 3101 (m, N–H stretch), 1674 (s, C=O stretch), 1335, 1157 (s, SO_2_ stretch); ^1^H NMR (400 MHz, DMSO-d_6_) δ (ppm): 8.03 (s, ex, 1H, OH/NH), 7.88 (dd, *J* = 6.8 Hz, *J* = 2.0 Hz, 2H, Ar), 7.58–7.54 (m, 5H, Ar, SO_2_NH_2,_ OH/NH), 7.42–7.37 (m, 5H, Ar); ^13^C NMR (100 MHz, DMSO-d_6_) δ (ppm): 162.00, 145.35, 139.71, 139.33, 138.48, 131.00, 130.02, 128.57, 127.26, 126.91, 126.24; HRMS (ESI-MS) *m/z* 366.0629 (M + Na)^+^, C_15_H_13_N_5_O_3_SNa^+^, calcd 366.0635.

#### 1-[4-(Aminosulfonyl)phenyl]-5-(4-methoxyphenyl)-1*H*-1,2,3-triazole-4-carboxamide (**6c**)

Yield 76%; white solid; mp: 280–282 °C; IR(KBr) (ν, cm^−1^): 3441, 3325, 3240, 3101 (m, N-H stretch), 2916 (m, –CH_3_ stretch), 1690 (s, C=O stretch), 1342, 1165 (s, SO_2_ stretch); ^1^H NMR (400 MHz, DMSO-d_6_) δ (ppm): 7.95 (s, 1H, OH/NH), 7.90 (dd, *J* = 6.8 Hz, *J* = 2.0 Hz, 2H, Ar), 7.58–7.53 (m, 5H, OH/NH, Ar, SO_2_NH_2_), 7.29 (d, *J* = 8.8 Hz, 2H, Ar), 6.94 (dd, *J* = 6.8 Hz, *J* = 2.0 Hz, 2H, Ar), 3.77 (s, 3H, –CH_3_); ^13^C NMR (100 MHz, DMSO-d_6_) δ (ppm): 161.64, 160.03, 144.77, 138.93, 138.69, 138.15, 131.98, 126.79, 126.33, 117.40, 113.60, 55.17; HRMS (ESI-MS) *m/z* 396.0754 (M + Na)^+^, C_16_H_15_N_5_O_4_SNa^+^, calcd 396.0743.

#### 1-[4-(Aminosulfonyl)phenyl]-5-(2-naphthyl)-1*H*-1,2,3-triazole-4-carboxamide (**6d**)

Yield 75%; white solid; mp: 278–280 °C; IR(KBr) (ν, cm^−1^): 3472, 3364, 3217, 3063 (m, N–H stretch), 1682 (s, C=O stretch), 1350, 1165 (s, SO_2_ stretch); ^1^H NMR (400 MHz, DMSO-d_6_) δ (ppm): 8.03 (d, *J* = 8.8 Hz, 2H, OH/NH, Ar), 7.97 (d, *J* = 8.0 Hz, 1H, Ar), 7.76 (d, *J* = 8.8 Hz, 2H, Ar), 7.62–7.36 (m, 10H, Ar, SO_2_NH_2,_ OH/NH); ^13^C NMR (100 MHz, DMSO-d_6_) δ (ppm): 161.10, 144.86, 140.91, 137.94, 137.25, 132.71, 131.17, 130.07, 129.54, 128.36, 127.09, 126.64, 126.30, 125.55, 125.09, 124.45, 123.58; HRMS (ESI-MS) *m/z* 416.0805 (M + Na)^+^, C_19_H_15_N_5_O_3_SNa^+^, calcd 416.0794.

### Synthesis of 4-[4-(hydrazinocarbonyl)-5-alkyl/aryl-1*H*-1,2,3-triazol-1-yl]benzenesulphonamide (**7a**)

General procedure: A solution of appropriate 1,2,3-triazolic ester **4a**–**4d** (1.93 mmol) and hydrazine hydrate (5.81 mmol) in ethanol (15 ml) was refluxed for 10–12 h. The reaction was monitored through thin-layer chromatography. After completion, reaction mixture was concentrated and allowed to cool to room temperature. Solid thus obtained was filtered and crystallised from EtOH:THF (1:1) to afford the desired compounds **7a**–**7d** in good yields.

#### 4-[4-(Hydrazinocarbonyl)-5-methyl-1*H*-1,2,3-triazol-1-yl]benzenesulphonamide (**7a**)

Yield 92%; white solid; mp: 216–218 °C; IR(KBr) (ν, cm^−1^): 3325, 3178, 3078, 3016 (m, N–H stretch), 1670 (s, C=O stretch), 1342, 1165 (s, SO_2_ stretch); ^1^H NMR (400 MHz, DMSO-d_6_) δ (ppm): 9.79 (s, ex, 1H, NH), 8.06 (dd, *J* = 6.8 Hz, *J* = 2.0 Hz, 2H, Ar), 7.88 (dd, *J* = 6.8 Hz, *J* = 2.0 Hz, 2H, Ar), 7.57 (s, 2H, SO_2_NH_2_), 4.50 (s, ex, 2H, NH_2_), 2.58 (s, 3H, –CH_3_); ^13^C NMR (100 MHz, DMSO-d_6_) δ (ppm): 160.00, 145.06, 137.79, 137.69, 136.40, 127.11, 125.72, 9.21; HRMS (ESI-MS) *m/z* 319.0576 (M + Na)^+^, C_10_H_12_N_6_O_3_SNa^+^, calcd 319.0590.

#### 4-[4-(Hydrazinocarbonyl)-5-phenyl-1*H*-1,2,3-triazol-1-yl]benzenesulphonamide (**7b**)

Yield 70%; white solid; mp: 206 °C; IR(KBr) (ν, cm^−1^): 3402, 3317, 3186, 3094 (m, N–H stretch), 1668 (s, C=O stretch), 1335, 1157 (s, SO_2_ stretch); ^1^H NMR (400 MHz, DMSO-d_6_) δ (ppm): 9.88 (s, ex, 1H, NH), 7.89 (dd, *J* = 6.8 Hz, *J* = 2.0 Hz, 2H, Ar), 7.58 (dd, *J* = 6.8 Hz, *J* = 2.0 Hz, 2H, Ar), 7.54 (s, 2H, SO_2_NH_2_), 7.42–7.38 (m, 5H, Ar), 4.49 (s, ex, 2H, NH_2_); ^13^C NMR (100 MHz, DMSO-d_6_) δ (ppm): 159.67, 145.35, 139.22, 138.69, 138.45, 130.93, 130.10, 128.64, 127.31, 126.82, 125.99; HRMS (ESI-MS) *m/z* 381.0640 (M + Na)^+^, C_15_H_14_N_6_O_3_SNa^+^, calcd 381.0745.

#### 4-[4-(Hydrazinocarbonyl)-5-(4-methoxyphenyl)-1*H*-1,2,3-triazol-1-yl]benzenesulphonamide (**7c**)

Yield 70%; white solid; mp: 170–172 °C; IR(KBr) (ν, cm^−1^): 3333, 3232, 3103 (m, N-H stretch), 1659 (s, C=O stretch), 1335, 1165 (s, SO_2_ stretch);^1^H NMR (400 MHz, DMSO-d_6_) δ (ppm): 9.80 (s, ex, 1H, NH), 7.91 (dd, *J* = 6.8 Hz, *J* = 2.0 Hz, 2H, Ar), 7.57 (d, *J* = 8.8 Hz, 2H, Ar), 7.53 (s, 2H, SO_2_NH_2_), 7.29 (d, *J* = 8.8 Hz, 2H, Ar), 6.94 (dd, *J* = 6.8 Hz, *J* = 2.0 Hz, 2H, Ar), 4.48 (s, ex, 2H, NH_2_), 3.76 (s, 3H, –CH_3_); ^13^C NMR (100 MHz, DMSO-d_6_) δ (ppm): 160.10, 159.34, 144.78, 138.46, 138.13, 138.08, 131.90, 126.85, 126.24, 117.22, 113.69, 55.20; HRMS (ESI-MS) *m/z* 389.1026 (M + H)^+^, C_16_H_16_N_6_O_4_SH^+^, calcd 389.1032.

#### 4-[4-(Hydrazinocarbonyl)-5-(2-naphthyl)-1*H*-1,2,3-triazol-1-yl]benzenesulphonamide (**7d**)

Yield 72%; white solid; mp: 156 °C; IR(KBr) (ν, cm^−1^): 3279, 3063 (m, N–H stretch), 1659 (s, C=O stretch), 1327, 1165 (s, SO_2_ stretch); ^1^H NMR (400 MHz, DMSO-d_6_) δ (ppm): 9.53 (s, ex, 1H, NH), 8.04 (d, *J* = 8.0 Hz, 1H, Ar), 7.98 (d, *J* = 8.0 Hz, 1H, Ar), 7.76 (d, *J* = 8.8 Hz, 2H, Ar), 7.63 (d, *J* = 7.2 Hz, 1H, Ar), 7.58–7.44 (m, 7H,Ar, SO_2_NH_2_), 7.35 (d, *J* = 8.4 Hz, 1H, Ar), 4.45 (s, ex, 2H, NH_2_); ^13^C NMR (100 MHz, DMSO-d_6_) δ (ppm): 159.26, 145.30, 140.74, 138.42, 137.31, 133.22, 131.73, 130.67, 130.19, 128.90, 127.66, 127.20, 126.84, 126.01, 125.61, 124.94, 123.88; HRMS (ESI-MS) *m/z* 431.0894 (M + Na)^+^, C_19_H_16_N_6_O_3_SNa^+^, calcd 431.0903.

### Synthesis of 4-(4-(hydroxymethyl)-5-methyl-1*H*-1,2,3-triazol-1-yl)benzenesulphonamide (**8a**–**8d**)

General procedure: Appropriate 1,2,3-triazolic ester **4a**–**4d** (3.22 mmol) was dissolved in dry tetrahydrofuran (20 ml) and a suspension of LiAlH_4_ (6.44 mmol) in dry tetrahydrofuran (5 ml) was slowly added under anhydrous conditions. After 20 min of reaction at 20 °C, reaction mixture was refluxed for 2 h. After completion of the reaction, an aqueous solution of HCl 1 N was added dropwise until a neutral pH. The reaction mixture was concentrated under reduced pressure; the residue was taken into ethyl acetate and washed with water and brine. The organic layer was dried over MgSO_4_ and concentrated under reduced pressure. The residue was recrystalised in ethanol.

#### 4-(4-(Hydroxymethyl)-5-methyl-1*H*-1,2,3-triazol-1-yl)benzenesulphonamide (**8a**)

Yield 55%; pale yellow solid; mp: 184 °C; IR(KBr) (ν, cm^−1^): 3495 (br, O–H stretch), 3294, 3186, 3086 (m, N–H stretch), 1336, 1157 (s, SO_2_ stretch); ^1^H NMR (400 MHz,DMSO-d_6_) δ (ppm): 8.05 (dd, *J* = 6.8 Hz, *J* = 2.0 Hz, 2H, Ar), 7.85 (dd, *J* = 6.8 Hz, *J* = 2.0 Hz, 2H, Ar), 7.58 (s, 2H, SO_2_NH_2_), 5.20 (t, ex, *J* = 5.6 Hz, 1H, OH), 4.59 (d, *J* = 5.6 Hz, 2H, CH_2_), 2.39 (s, 3H, CH_3_); ^13^C NMR (100 MHz,DMSO-d_6_) δ (ppm): 145.71, 144.96, 139.01, 132.34, 127.65, 125.56, 54.68, 8.95.

#### 4-(4-(Hydroxymethyl)-5-phenyl-1*H*-1,2,3-triazol-1-yl)benzenesulphonamide (**8b**)

Yield 68%; pale yellow solid; mp: 174–176 °C; IR(KBr) (ν, cm^−1^): 3325 (br, O–H stretch), 3225, 3171, 3070 (m, N–H stretch), 1342, 1157 (s, SO_2_ stretch); ^1^H NMR (400 MHz, CDCl_3_) δ (ppm): 7.97 (d, *J* = 8.8 Hz, 2H, Ar), 7.48–7.44 (m, 5H, Ar, SO_2_NH_2_), 7.37–7.35 (m, 2H, Ar), 7.28 (d, *J* = 7.2 Hz, 2H, Ar), 5.25 (s, ex, 1H, OH), 4.65 (s, 2H, CH_2_); ^13^C NMR (100 MHz, CDCl_3_) δ (ppm): 150.68, 149.26, 143.74, 140.40, 134.54, 134.33, 133.81, 132.11, 131.06, 129.72, 59.36.

#### 4-(4-(Hydroxymethyl)-5-(4-methoxyphenyl)-1*H*-1,2,3-triazol-1-yl)benzenesulphonamide (**8c**)

Yield 69%; yellow solid; mp: 180–182 °C; IR(KBr) (ν, cm^−1^): 3481 (br, O–H stretch), 3295 (m, N–H stretch), 2916 (m, –CH_3_ stretch), 1342, 1142 (s, SO_2_ stretch); ^1^H NMR (400 MHz, CDCl_3_) δ (ppm): 7.99–7.95 (m, 2H, Ar), 7.48–7.44 (m, 2H, Ar), 7.30–7.25 (m, 4H, Ar, SO_2_NH_2_), 6.96–6.92 (m, 2H, Ar), 5.22 (t, *J* = 5.6 Hz, 1H, OH), 4.63 (d, *J* = 5.6 Hz, 2H, CH_2_), 3.85 (s, 3H, CH_3_); ^13^C NMR (100 MHz, CDCl_3_) δ (ppm): 165.14, 150.34, 149.16, 143.87, 140.36, 135.92, 132.09, 129.70, 122.94, 119.31, 60.14, 59.41.

#### 4-(4-(Hydroxymethyl)-5-(2-naphthyl)-1*H*-1,2,3-triazol-1-yl)benzenesulphonamide (**8d**)

Yield 71%; dirty white solid; mp: 212–214 °C; IR(KBr) (ν, cm^−1^): 3431 (br, O–H stretch), 3295 (m, N–H stretch), 1335, 1157 (s, SO_2_ stretch); ^1^H NMR (400 MHz, CDCl_3_) δ (ppm): 8.02 (dd, *J* = 6.8 Hz, *J* = 2.4 Hz, 1H, Ar), 7.94 (d, *J* = 6.0 Hz, 1H, Ar), 7.76 (d, *J* = 8.4 Hz, 2H, Ar), 7.58–7.57 (m, 2H, Ar), 7.53–7.49 (m, 1H, Ar), 7.42–7.38 (m, 4H, Ar), 7.27 (s, 2H, SO_2_NH_2_), 5.11 (t, *J* = 5.2 Hz, 1H, OH), 4.48 (ddd, *J* = 58.4 Hz, *J* = 12.4 Hz, *J* = 5.2 Hz, 2H, CH_2_); ^13^C NMR (100 MHz, CDCl_3_) δ (ppm):152.24, 149.19, 143.80, 138.52, 138.23, 136.38, 135.34, 134.64, 133.53, 132.28, 131.96, 131.53, 130.37, 129.40, 128.73, 128.67, 59.26.

## Results and discussion

### Chemistry

**Scheme 1.**
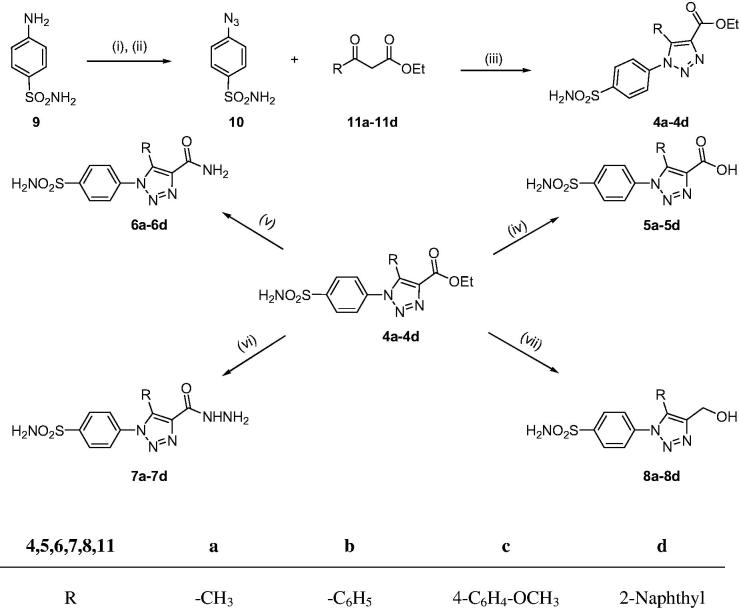
Synthetic pathway to the sulphonamides **4a**–**4d**, **5a**–**5d**, **6a**–**6d**, **7a**–**7d**, and **8a**–**8d**. Reagents and conditions: (i) HCl, NaNO_2_, H_2_O, 0 °C, 15 min; (ii) NaN_3_, 0 °C, 30 min; (iii) Piperidine, DMSO, 70 °C, 4 h; (iv) NaOH, reflux, 3 h then H_3_O^+^; (v) NH_3_ solution, stir, 22 h; (vi) NH_2_NH_2_.H_2_O, EtOH, reflux, 10–12 h; (vii) LiAlH_4_, dry THF, reflux, 2 h then H_3_O^+^.

The synthetic route adopted for the synthesis of 4-functionalised 1,2,3-triazole compounds (**4a**–**4d**, **5a**–**5d**, **6a**–**6d**, **7a**–**7d**, and **8a**–**8d**) is outlined in Scheme 1. 1,2,3-Triazole-4-carboxylates **4a**–**4d**, the supreme compounds to carry out the complete conversion, were synthesised by reactions of 4-azidobenzenesulphonamide (**10**) with differently substituted β-ketoesters (**11a**–**11d**) in the presence of organic base. 4-Azidobenzenesulphonamide (**10**) in turn was prepared from sulphanilamide (**9**) via diazotisation followed by treatment with sodium azide^47^. After the synthesis, 1,2,3-triazole-4-carboxylates **4a**–**4d** were converted to corresponding carboxylic acids **5a**–**5d** by hydrolysis with a strong base and corresponding carboxamide derivatives **6a**–**6d** by treatment with ammonia solution. 1,2,3-Triazole-4-hydrazinocarbonyl derivatives **7a**–**7d** were obtained by treating their corresponding esters **4a**–**4d** with hydrazine hydratein ethanol while 1,2,3-triazole-4-hydroxymethyl derivatives **8a**–**8d** were prepared by treating esters **4a**–**4d** with lithium aluminium hydride^48^ (Scheme 1).

The structures of all the newly synthesised compounds (**4a**–**4d**, **5a**–**5d**, **6a**–**6d**, **7a**–**7d**, and **8a**–**8d**) were characterised by a rigorous analysis of their IR, ^1^H NMR and ^13^C NMR spectral data. Structures were further confirmed by their HRMS data. In FT-IR, a strong characteristic absorption band for C=O stretch was observed in the range 1704–1728 cm^−1^ for 1,2,3-triazole-4-carboxylates **4a**–**4d**, 1690–1713 cm^−1^ for 1,2,3-trazole-4-carboxylic acids **5a**–**5d**, 1674–1690 cm^−1^ for 1,2,3-triazole-4-carboxamides **6a**–**6d** and 1659–1670 cm^−1^ for 1,2,3-triazole-4-hydrazinocabonyl derivatives **7a**–**7d** while no such absorption band was observed in 1,2,3-triazole-4-hydroxymethyl compounds **8a**–**8d** showing the complete reduction of ester group to hydroxymethyl group. The NMR spectra of ethyl esters of 1,2,3-triazole-4-caboxylic acids **4a**–**4d** displayed a quartet in the range δ 4.37–4.00 ppm of two protons and a triplet in the range δ 0.81–1.35 ppm of three protons for ethyl group. Conversion of ester compounds **4a**–**4d** to the corresponding carboxylic acids **5a**–**5d** was confirmed by a downfield exchangeable singlet around δ 13.00 ppm due to COOH with disappearance of signals due to ethyl group protons. Similarly, 1,2,3-triazole-4-carboxamides **6a**–**6d** were characterised by two exchangeable singlets in the range of δ 7.94–8.04 ppm and 7.52–7.54 ppm corresponding to NH and OH protons, target 1,2,3-triazole-4-hydrazinocarbonyls **7a**–**7d** showed two exchangeable singlets in the range δ 9.53–9.88 ppm due to NH proton and δ 4.45–4.50 ppm due to NH_2_ protons whereas 1,2,3-triazole-4-hydroxymethyl compounds **8a**–**8d** displayed a triplet around δ 5.11–5.25 ppm of OH and a doublet around δ 4.48–4.65 ppm of CH_2_ protons. The presence of sulphonamide group in all the target 4-functionalised 1,2,3-triazole compounds (**4**–**8**) was evident from a broad singlet, exchangeable in D_2_O, appearing in the range δ 7.44–7.56 ppm.

### CA inhibition

All the newly synthesised 4-functionalised 1,2,3-triazole compounds (**4a**–**4d**, **5a**–**5d**, **6a**–**6d**, **7a**–**7d**, and **8a**–**8d**) were evaluated against cytosolic isoenzymes hCA I & hCA II and membrane bound isoenzymes hCA IV & hCA IX for their CA inhibition potential by using stopped-flow CO_2_hydrase assay method^49^ and acetazolamide (**AZA**) was chosen as reference drug for the assay. In general, all the assayed compounds have shown significant inhibitory action against the reported isoforms, with low nanomolar inhibition constant (Ki). Inhibition data of the compounds as given in Table 1 let the following insights regarding CAs inhibitory properties.

**Table 1.** Inhibitory potency data for compounds **4a**–**4d**, **5a**–**5d**, **6a**–**6d**, **7a**–**7d**, and **8a**–**8d** against isozymes hCAI, hCA II, hCA IV, and hCA IX.

Compound	R	Ki (nM)^a^			
		hCA I	hCA II	hCA IV	hCA IX
**4a**	–CH_3_	9.1	3.2	3.5	6.6
**4b**	–C_6_H_5_	245.5	6	5.1	9.5
**4c**	4-C_6_H_4_–OCH_3_	63	6.1	4.5	40
**4d**	2-Naphthyl	68.2	5.8	5	7.1
**5a**	–CH_3_	9.6	1.6	269.4	8.5
**5b**	–C_6_H_5_	645.7	97.9	55.3	45.9
**5c**	4-C_6_H_4_–OCH_3_	489.1	9	2.4	42.3
**5d**	2-Naphthyl	56.2	8.9	4.1	4.2
**6a**	–CH_3_	8.3	1.9	211.9	9.5
**6b**	–C_6_H_5_	69.1	9.1	1.4	28.3
**6c**	4-C_6_H_4_–OCH_3_	873.7	6.2	1.7	6.8
**6d**	2-Naphthyl	771.7	6	4	6.7
**7a**	–CH_3_	15.1	7.6	227.8	9.3
**7b**	–C_6_H_5_	73.7	38	22.7	26.1
**7c**	4-C_6_H_4_–OCH_3_	91.2	5.6	4	8.1
**7d**	2-Naphthyl	82.5	1.6	2	7.2
**8a**	–CH_3_	354.2	58.9	359.6	30.6
**8b**	–C_6_H_5_	78.9	9.4	8.4	58.7
**8c**	4-C_6_H_4_–OCH_3_	811.8	4	1.9	5.8
**8d**	2-Naphthyl	213.5	8.1	1.9	7.8
**AZA**		250	12.1	74	25.8

AZA: acetazolamide (reference compound).

a Mean from three different assays, by a stopped flow technique (errors were in the range of ±5–10% of the reported values).

- The cytosolic isoform hCA I was in general significantly inhibited by all the newly synthesised compounds (**4a**–**4d**, **5a**–**5d**, **6a**–**6d**, **7a**–**7d**, and **8a**–**8d**) with Ki in the range 8.3 nM–0.8737 µM (Table 1). It is pertinent to mention that 5-CH3 substituted derivatives of newly synthesised compounds except **8a** were most effective inhibitors of hCA I with Ki ≤ 15.1nM as compared to corresponding 5-aryl derivatives. At the same time some compounds namely **5b**, **5c**, **6c**, **6d**, **8a**, and **8c** showed weaker inhibition potential as compared to reference drug AZA (Ki =250 nM) against hCA I that is off-target while inhibiting hCA II and IV in glaucoma and hCA IX in tumours.
- Nearly all the newly synthesised compounds (**4a**–**4d**, **5a**–**5d**, **6a**–**6d**, **7a**–**7d**, and **8a**–**8d**) showed better inhibitory potential in low nanomolar range with Ki ≤ 9.4 nM except three compounds namely **5b**, **7b**, and **8a** against the most abundant isoform hCA II as compared to standard drug AZA (Ki =12.1 nM). Some of the tested compounds mainly 5-CH3 derivatives (**4a**, **5a**, and **6a**) and two other compounds (**7d** and **8c**) exhibited inhibitory potency (Ki ≤ 4 nM) several times better than AZA (Table 1).
- All the tested compounds except some 5-CH3 derivatives namely **5a**, **6a**, **7a**, and **8a** showed excellent inhibition potential with Ki in the range of 1.4–55.3 nM against membrane bound isoform, hCA IV as compared to standard drug AZA. Most of the compounds (**4a**–**4d**, **5c**–**5d**, **6b**–**6d**, **7c**–**7d**, and **8b**–**8d**) have their inhibitory potency (Ki ≤ 8.4 nM) several folds superior than AZA (Ki ≤ 74 nM) against hCA IV which is one of the drug target for designing antiglaucoma drugs (Table 1).
- In general, all the tested compounds except few (**4c**, **5b**, **5c**, **6b**, **7b**, **8a**, and **8b**) have shown better activity profile (Ki ≤ 9.5 nM) against tumour associated membrane bound isozyme hCA IX as compared to reference drug AZA (Ki =25.8 nM). It is significant to mention here that, in the broader sense, derivatives with 5-CH3and 5-(naphtha-2-yl) substitution have shown better activity as compared to other derivatives (Table 1).
- Interestingly compounds possessing rather bulky scaffolds were milder inhibitors of cytosolic isoform hCA I, over other isoforms (hCA II, IV, and IX) and is mainly due to the fact that the active site cavity of hCA I is smaller than other isozymes hCA II, IV and IX, because of the presence of two His residues (i.e. His 200 and His 67)^50^. Overall comparison of activity, in terms of SAR, reveals that all the compounds except derivatives with 5-CH3 group were better selective for hCA II and IV over hCA I in the broader sense. Therefore, these compounds can be important candidates for designing antiglaucoma drugs. However, their good activity against both hCA II and hCA IX suggests that structure of compounds needs further modification for getting better selectivity for tumour associated hCA IX over hCA II.

## Conclusions

In this paper, we report a series of twenty novel compounds of 4-functionalised 1-aryl-5-alkyl/aryl-1,2,3-triazole compounds (**4a**–**4d**, **5a**–**5d**, **6a**–**6d**, **7a**–**7d**, and **8a**–**8d**) bearing a primary sulphonamide group on the phenyl ring at N-1 position of 1,2,3-triazole scaffold with different functionalities at C-4, such as ester, carboxylic acid, carboxamide, hydrazinocarbonyl, and hydroxymethyl which were evaluated against four isozymes, hCA I, II, IV, and IX. Most of the compounds performed better against aforementioned isoforms showing low nanomolar potency as compared to reference drug acetazolamide. Out of twenty newly synthesised compounds, seventeen compounds (except **5 b**, **7 b**, and **8a**) showed low nanomolar affinity (Ki ≤ 9.4 nM) for hCA II, sixteen compounds except the derivatives with 5-CH_3_ substitution have displayed excellent activity (Ki ≤ 55.3 nM) for hCA IV and thirteen compounds (except **4c**, **5 b**, **5c**, **6 b**, **7 b**, **8a**, and **8 b**) have shown better activity (Ki ≤ 9.5) for hCA IX while most of compounds with bulkier substitution at C-5 were medium to weaker inhibitors of hCA I with Ki values in the range of 56.2–873.7 nM. In short, reported compounds have shown remarkable activity against hCA I, II, IV, and IX isoforms from which it can be concluded that 1,2,3-triazoles scaffold deserve to be investigated further as a novel scaffold for CAIs.
